# Cytotoxicity of glass ionomer cements containing silver nanoparticles

**DOI:** 10.4317/jced.52566

**Published:** 2015-12-01

**Authors:** Patrícia-Correia Siqueira, Ana-Paula-Rodrigues Magalhães, Wanessa-Carvalho Pires, Flávia-Castro Pereira, Elisângela-Paula Silveira-Lacerda, Marcus-Santos Carrião, Andris-Figueiroa Bakuzis, Carlos-Alberto Souza-Costa, Lawrence-Gonzaga Lopes, Carlos Estrela

**Affiliations:** 1 DDS, MSc. Department of Stomatological Sciences, School of Dentistry, Federal University of Goiás, Goiânia, Goiás, Brazil; 2 DDS, MSc. Department of Prevention and Oral Rehabilitation, School of Dentistry, Federal University of Goiás,Goiânia, Goiás, Brazil; 3 MSc. Laboratory of Molecular Genetics and Cytogenetics, Federal University of Goiás,Goiânia, Goiás, Brazil; 4 MSc, PhD. Laboratory of Molecular Genetics and Cytogenetics, Federal University of Goiás, Goiânia, Goiás, Brazil; 5 MSc. Physics Institute, Federal University of Goiás, Goiânia, Goiás, Brazil; 6 MSc, PhD. Physics Institute, Federal University of Goiás, Goiânia, Goiás, Brazil; 7 DDS, MSc, PhD. Department of Physiology and Pathology, School of Dentistry (UNESP), Araraquara, São Paulo, Brazil; 8 DDS, MSc, PhD. Department of Prevention and Oral Rehabilitation, School of Dentistry, Federal University of Goiás, Goiânia, Goiás, Brazil; 9 DDS, MSc, PhD. Department of Stomatological Sciences, School of Dentistry, Federal University of Goiás, Goiânia, Goiás, Brazil

## Abstract

**Background:**

Some studies have investigated the possibility of incorporating silver nanoparticles (NAg) into dental materials to improve their antibacterial properties. However, the potential toxic effect of this material on pulp cells should be investigated in order to avoid additional damage to the pulp tissue. This study evaluated the cytotoxicity of conventional and resin-modified glass ionomer cements (GIC) with and without addition of NAg.

**Material and Methods:**

NAg were added to the materials at two different concentrations by weight: 0.1% and 0.2%. Specimens with standardized dimensions were prepared, immersed in 400 µL of culture medium and incubated at 37°C and 5% CO2 for 48 h to prepare GIC liquid extracts, which were then incubated in contact with cells for 48 h. Culture medium and 0.78% NAg solution were used as negative and positive controls, respectively. Cell viability was determined by MTT and Trypan Blue assays. ANOVA and the Tukey test (α=0.05) were used for statistical analyses.

**Results:**

Both tests revealed a significant decrease in cell viability in all groups of resin modified cements (*p*<0.001). There were no statistically significant differences between groups with and without NAg (*p*>0.05). The differences in cell viability between any group of conventional GIC and the negative control were not statistically significant (*p*>0.05).

**Conclusions:**

NAg did not affect the cytotoxicity of the GIC under evaluation.

** Key words:**Glass ionomer cements, totoxicity, cell culture techniques, nanotechnology, metal nanoparticles.

## Introduction

In treatments to restore dental function and improve esthetics, operative and restorative procedures should respect the biological principles of vital pulps (1). Besides bacteria and their toxic products, dental materials applied to the dentin-pulp complex may also release cytopathic components that diffuse through the dentinal tubules to cause pulpal damage (2).

The dentin-pulp complex should be protected to preserve the viability of the dental pulp, a connective tissue that has an inherent capacity to respond defensively against aggressive stimuli (1). One or more layers of specific biocompatible material should be placed between the restorative material and the dental tissue to avoid additional damage to the pulp due to operative procedures, exposure to toxic restorative materials or bacterial microleakage (2). Different materials have been suggested to protect the den-tin-pulp complex, which present biological tolerance and prevent or limit pulp irritation (2). Some of the materials indicated are glass ionomer cements (GIC), whose use has been growing because of their properties: adhesion to tooth structure, fluoride release and tissue tolerance (3).

Regardless of the material selected to be placed on the bottom of deep cavities, the pulp is capable of repair as long as microbial contamination is prevented (4). It is known that bacteria may still be present on dentin after cavity restoration if the tissue affected by caries has not been fully removed or if there is microleakage (5,6). Then, bacterial contamination may increase under the restoration and induce the development of secondary caries, which reduces restoration longevity and leads to pulp damage (7).

The antibacterial properties of GIC result from its release of fluoride, which reduces demineralization, stimulates remineralization, reduces biofilm formation and inhibits bacterial growth and metabolism (3,7). Studies have verified ways to improve the antibacterial activity of these materials by combining them with antimicrobial agents to reduce the occurrence of secondary caries (6,8,9).

Silver has been studied in several areas (10-12) because of its broad spectrum of antimicrobial activity against Gram-positive and negative bacteria, fungi, protozoa and some viruses (11,12). Nanotechnology has made it possible to better explore the antimicro-bial properties of silver, used as nanoparticles. Silver particles have different applications in cosmetics, textile industry, water purification systems, catheter coatings and wound dressings (13). Silver nanoparticles (NAg) are insoluble clusters of silver atoms measuring less than 100nm (11,12). Their size is an important characteristic because smaller particles produce higher specific surface areas and, therefore, reduce particle concentration necessary for efficacy (10,14).

Recent studies have investigated the possibility of incorporating silver nanoparticles into dental materials to improve their antibacterial properties (14-16). However, the potential toxic effect of NAg on pulp cells, which may be in direct or indirect contact with material that contains these nanoparticles, should be investigated. Few studies have evaluated the effect of NAg on the cytotoxicity of dental materials (14,16), but some studies have reported on the cytotoxic and genotoxic effects of NAg (13,17-19). This study evaluated the cytotoxicity of a conventional GIC and a resin modified GIC with and without the addition of NAg using an odontoblast cell line.

## Material and Methods

Two types of GIC indicated for cavity lining were used in this study: a conventional GIC (GC Gold Label 1, GC Corporation, Tokyo, Japan) and a resin modified GIC (Vitrebond, 3M ESPE Dental Products, St. Paul, MN, USA).  Table 1 shows the characteristics of each cement. The research protocol was performed according to a previous study (6).

**Table 1:**
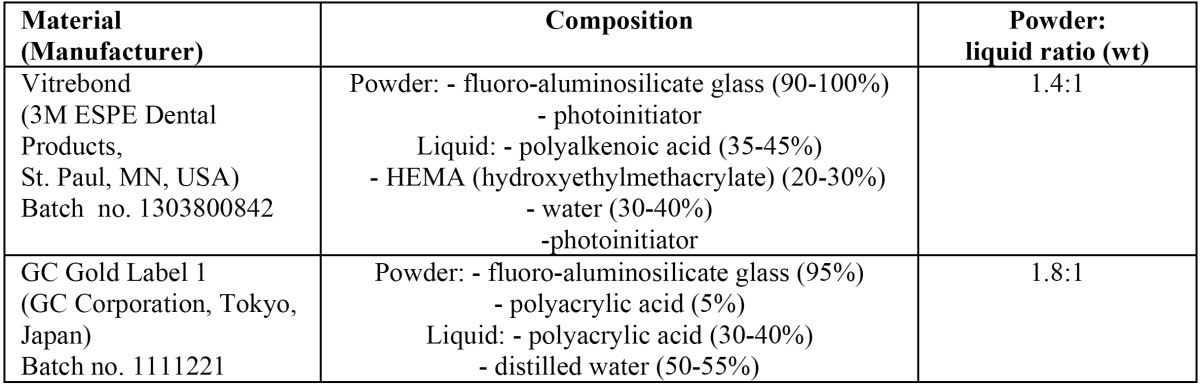
Description of the glass ionomer cements.

-Synthesis of silver nanoparticles

Silver nanoparticles (NAg) were prepared in the Physics Institute of Federal University of Goiás (Goiânia, Brazil) by adding sodium borohydride (0.002 M) (Sigma-Aldrich, St. Louis, MI, USA) to reduce silver nitrate (0.001 M) (Sigma-Aldrich, St. Louis, MI, USA) at a controlled low temperature and using a magnetic stirrer (TE 080, Techal, São José dos Campos, SP, Brazil). The chemical equation below describes this method: AgNO3 + NaBH4→ Ag + ½ H2 +1/2 B2H6 + NaNO3.

To destabilize the solution and stimulate NAg precipitation, 3.0 mL of 0.75 M sodium chloride (Sigma-Aldrich, St. Louis, MI, USA) was added. Then, the solution was concentrated to obtain an aqueous solution at 0.78%. The final solutions were shaken in an ultrasonic bath (USC-2800, Unique, Indaiatuba, SP, Brazil) for three cycles of 10 minutes to disperse the metal completely in the solution.

-Preparation of specimens

The cements were prepared according to their manufacturers’ specifications, and silver was either added or not, according to the group to which the specimen belonged. The NAg solution was incorporated into the material during preparation to obtain specimens with two concentrations of silver: 0.1% and 0.2%. The powder was weighed on an analytical scale, and specimens were prepared according to weight.

Specimens of each type of cement were divided into three groups: cement without NAg; cement with 0.1% NAg; and cement with 0.2% NAg. Three specimens were prepared for each group. Cement specimens were then placed in a circular stainless steel mold (2.0 mm thick, 4.0 mm diameter) (6). The conventional GIC (GC Gold Label 1) specimens were kept in the mold for 10 minutes to set. The resin modified GIC (Vitrebond) specimens were light-cured using a light-emitting diode unit (LED) (Emitter, Schuster, Santa Maria, RS, Brazil) and a continuous polymerization technique (600 mW/cm²) applied for 40 s to each side.

The material and specimens were prepared under laminar flow. After the setting, the specimens were removed from the mold and exposed to ultraviolet (UV) radiation for 40 minutes on each side to prevent contamination during the tests (20).

-Extract preparation

To simulate the indirect contact of the cells with the cements, liquid extracts of the cements were prepared as recommended by ISO 10993-5. To obtain the extracts, specimens were placed separately in microtubes (Eppendorf Ltda., São Paulo, Brazil) with 400 µL of Dulbecco’s modified eagle’s medium (DMEM) supplemented with 10% fetal bovine serum (FBS) at a 1.25 cm2/mL ratio of surface area/volume. Medium and specimens immersed in medium were kept in a humidified incubator at 37 °C and 5% CO2 for 48 hours. After that, the specimens were removed from the microtubes, and the extracts were used for toxicity testing.

-Cell viability assays

The cytotoxicity tests were performed with the odontoblast-like MDPC-23 cells, which have been widely used in dentistry (21). Cells were seeded at a density of 3x104 cells/cm2 in a 96-well plate containing 200 µL of supplemented culture medium in each well. The plates were kept in a humidified incubator at 37 °C and 5.0% CO2 for 24 hours. After that, the culture medium was discarded and 180 µL of supplemented culture medium and 20 µL of cement extracts were added to each well at a 10% dilution of the original extracts.

Supplemented culture medium was used as negative control (DMEM + 10% fetal bovine serum) and 0.78% NAg solution, as positive control. For the negative control, 200 µL of culture medium were used, and for the positive control, 20 µL of the NAg solution and 180 µL of culture medium. The treated cells were incubated for 48 hours. All experiments were performed in triplicate, with each experiment in three wells, at nine wells for each material.

To assess cell metabolism, a methyltetrazolium (MTT) colorimetric assay was used. After the incubation of the cells with the extracts, the culture medium in the wells was discarded, and 40 µL of phosphate buffered saline (PBS) and 10 µL of MTT (5.0 mg/mL) were added. After 3 hours of incubation with MTT, 50 µL of sodium dodecyl sulfate (SDS) was added to interrupt MTT reaction. A spectrophotometer (INE Awareness Technology, Palm City, FL, USA) was used to measure optical density, and the values were used to calculate cell viability percentages.

A Trypan Blue assay was performed to assess cell integrity at membrane level. After cell incubation with the extracts, the culture medium was discarded, washed with DMEM, and 150 µL of trypsin and ethylenediaminetetraacetic acid (EDTA) was added to each well. The plate was incubated for 5 minutes, and then 150 µL of supplemented culture medium was added. The contents of each well were aspirated, placed into labeled microtubes and centrifuged at 1500 rpm for 10 minutes. The supernatant was discarded, and the cells were suspended again in 100 µL of culture medium. An aliquot of 10 µL of cell suspension was removed and mixed with 10 µL of trypan blue. After homogenization, the live and dead cells were counted using an electronic counter (Luna, Logos Biosystems, Annandale, VA, USA), and the percentage of viable cells was calculated.

-Statistical analysis

Test results were described as means and standard deviations. As variables had a normal distribution (Kolmogorov-Smirnov), ANOVA was used to evaluate the effect of the different variables, and the Tukey test, for multiple comparisons. GraphPad Prism 4.0 was used for statistical analysis, and the level of significance was set at 5% (α=0.05).

## Results

 Table 2 shows cell viability percentages determined by MTT and Trypan Blue assays. For the negative control, mean cell viability determined by MTT assay was 100.6%, and by Trypan Blue staining, 93.2%.

**Table 2:**
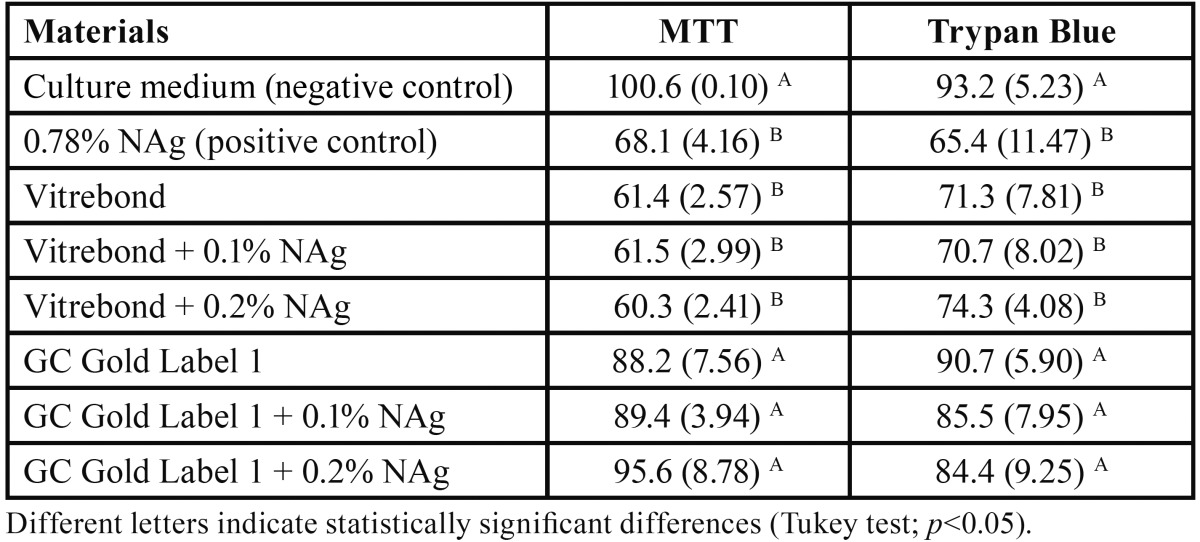
Cell viability percentages (mean and standard deviation) determined by MTT and Trypan Blue assays.

Cell viability determined by both assays decreased significantly in the Vitrebond group with no NAg when compared with the negative control (*p*<0.001): mean cell viability determined by MTT was 61.4%, and by TrypanBlue assay, 71.3%. Viability also decreased significantly in all Vitrebond groups with NAg, regardless of concentration (*p*<0.001). There were no significant differences between groups with different NAg concentrations (*p*>0.05) or between groups with and without NAg (*p*>0.05).

There were no significant differences in cell viability determined by either test between any of the GC Gold Label 1 groups (no NAg; 0.1% NAg; 0.2% NAg) and the negative control (*p*>0.05). Similarly, there were no significant differences between the groups with and without NAg (*p*>0.05).

The comparison of the positive control group (0.78% NAg) with the negative control revealed a significant decrease in cell viability (*p*<0.001): mean cell viability determined by MTT was 68.1%, and by Trypan Blue, 65.4%. However, there were no significant differences between the positive control and the Vitrebond groups (with and without NAg) (*p*>0.05).

**Figure 1:**
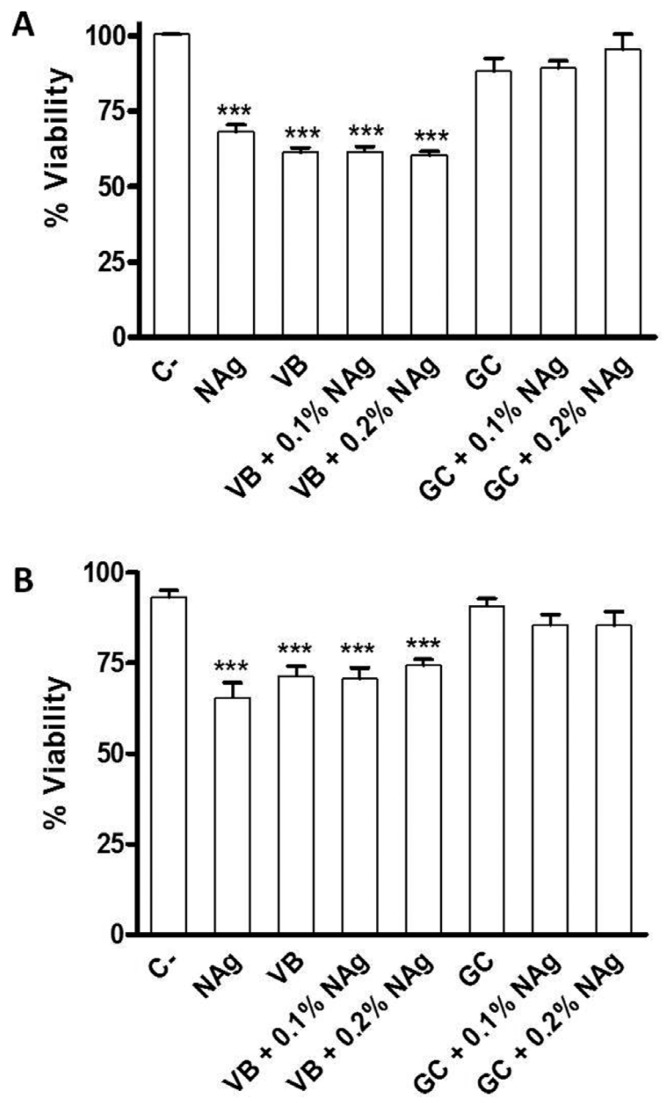
Distribution of cell viability percentages determined by MTT A) and Trypan Blue B) assays according to tested materials (ANOVA and Tukey test). C-: negative control; VB: Vitrebond; GC: GC Gold Label 1. *** *p*<0.001 compared with negative control.

Figure 1 shows the distribution of cell viability percentages determined by MTT and trypan blue assays in each group.

## Discussion

The cytotoxicity of dental materials placed on the bottom of a deep cavity should be fully understood because of the possibility of indirect contact between their components and pulp cells (1). Residual components released from these materials may diffuse through the dentinal tubules and reach the subjacent odontoblast layer (22). For this reason, cultures of odontoblast-like cells have been widely used to evaluate the cytotoxic effects of various dental materials (21,22). Then, immortalized odontoblast-like MDPC-23 cells that have phenotypic markers of odontoblasts and synthesize proteins of the dentinal matrix were used in the present study (21).

Two viability assays were employed in this investigation. The MTT assay determines cell viability based on the activity of the dehydrogenase enzyme, found in mitochondria and associated with cell metabolism. The Trypan Blue assay assesses the cell viability according to cell membrane integrity, as viable cells are not permeable to this dye. Both assays yielded similar results in the comparison of the groups under study.

The percentages of cell viability found for the conventional GIC (GC Gold Label 1) were similar to those of the negative control (*p*>0.05). Therefore, this material was not toxic to the MDPC-23 cells. Although no other studies have evaluated the cytotoxicity of this specific cement, conventional GICs are known to have good tissue tolerance, particularly when compared with resin modified GICs (3,23,24). GIC powder contains glass particles (SiO2, Al2O3, CaF2, Na3AlF6, AlF3, AlPO4), and metal ions (Al3+, Na+ e Ca2+) are dissolved from powder to liquid during setting. In the same way as silica (SiO2), the basic substance of glass powder, these metal ions are not toxic or irritating when in contact with living cells (24).

The resin modified GIC (Vitrebond) evaluated in this study had a defined toxic effect on MDPC-23 cells, with a significant reduction of cell viability even without the addition of NAg on its composition. This result is in agreement with previous studies in which the authors showed that Vitrebond is cytotoxic, causes intense morphological changes and cell death (25-26).

The incorporation of resin components, such as monomers and photoinitiators, into conventional GIC formulations has been associated with their increased cytotoxic effects (25). Residual HEMA, because of its hydrophilic properties and low molecular weight, may easily diffuse through dentinal tubules and reach pulp cells (24). Recent studies also found strong correlations between fluoride release and the cytotoxic effects of GICs (26,27). The authors showed that the most cytotoxic GICs released higher amount of fluoride compared to other materials which leached small amounts of fluoride and consequently caused slight toxicity. In both studies, Vitrebond was the GIC that released the largest amount of fluoride ions, which may be a determinant factor in the cytotoxicity of this material (26,27).

The attempt to improve the antimicrobial properties of dental materials has led researchers to evaluate antimicrobial agents that may be added to materials without adversely affecting their physical and mechanical properties (14-16). The use of NAg in medicine and dentistry has been encouraged because it has a broad-spectrum antimicrobial effect when used at low concentrations, and because it does not lead to the development of resistant bacterial strains (10,16). The antimicrobial mechanism of NAg has not been fully elucidated (12), but is probably associated with the release of silver ions, formation of reactive oxygen species (ROS) and direct interaction of the particles with the membrane of microorganisms (10,12). However, cytotoxic effects of NAg have been found not only in microbial cells, but also in human cells. Studies found an association of these cytotoxic effects with the induction of dose- and time-dependent ROS formation, which leads to cell death (13,17).

In the present study, the cytotoxic effect of the NAg solution (positive control) on the odontoblast-like MDPC-23 cells led to a significant decrease in cell viability after 48 hours of exposure. Similar cytotoxicity results have been found in previous studies (13,17,18,28). AshaRani *et al.* (28) evaluated the cytotoxicity and genotoxicity of NAg and found that the nanoparticles reduced adenosine triphosphate (ATP) in the cells and affected mitochondria and ROS production in a dose-dependent manner. DNA damage was also dose-dependent and the scanning electron microscopy assessment of cells exposed to NAg revealed the presence of particles inside mitochondria and nuclei, which confirmed its direct involvement in mitochondrial toxicity and DNA damage. Based on these findings, a possible mechanism of NAg cytotoxicity has been described: NAg particles disrupt the mitochondrial respiratory chain, leading to ROS production and interrupting ATP synthesis, which, in turn, results in DNA damage (28).

In addition to dose and exposure time, particle size may also play a role in the cytotoxic effect of NAg, and smaller particles seem to have a more toxic effect (13). Park *et al.* (13) compared the cytotoxicity of particles of different diameters and found that smaller particles (20 nm) were more cytotoxic than larger particles (80 nm to 110 nm). In the present study, the method used for NAg synthesis produced 12 ± 2 nm particles, which may explain their cytotoxicity. Although NAg was cytotoxic to the cells in this study, their incorporation did not affect the cytotoxicity of the GICs. Both Vitrebond and GC Gold Label 1 groups with NAg (0.1% and 0.2%) had no significant differences in cell viability from the groups without NAg.

Recent studies (14,16) evaluated the effect of NAg on the cytotoxicity of dental materials and, specifically, the cytotoxicity of adhesives and primers containing NAg. Their results were similar to our findings in that the addition of NAg to dental adhesives at concentrations of 0.05% and 0.1% did not affect cytotoxicity. In the same way, Zhang *et al.* (16) reported that the addition of NAg to primers at a concentration of 0.05% did not affect cytotoxicity in cultured fibroblasts. These results may be explained by the fact that the low concentration of NAg added to materials may not affect cytotoxicity. Despite the satisfactory results of these in vitro trials, further studies, both in vitro and in vivo, should be conducted to confirm the biological safety of adding NAg to dental materials, such as glass-ionomer cements and other resin-modified products.

According to the methodology used in this study, the resin modified GIC (Vitrebond) causes significant cytotoxicity to the cultured odontoblast-like MDPC-23 cells, whereas the conventional GIC (GC Gold Label 1) was not toxic. Addition of silver nano-particles to both materials does not affect their cytotoxic effects.
